# Artificial intelligence-derived transition zone PSA density as a triage tool to reduce unnecessary prostate systematic biopsies in MRI-negative men

**DOI:** 10.1186/s13244-026-02221-8

**Published:** 2026-02-10

**Authors:** Jiaheng Shang, Jingyun Wu, Ruiyi Deng, Meixia Shang, Pengsheng Wu, Jianhui Qiu, Jingcheng Zhou, Lin Cai, Xiaoying Wang, Kan Gong, Yi Liu

**Affiliations:** 1 Department of Urology, Peking University First Hospital, Beijing, China; 2 Institute of Urology, Peking University, Beijing, China; 3 National Urological Cancer Center, Beijing, China; 4 Department of Radiology, Peking University First Hospital, Beijing, China; 5 Department of Biostatistics, Peking University First Hospital, Beijing, China; 6 Beijing Smart Tree Medical Technology Co. Ltd., Beijing, China

**Keywords:** Negative MRI, Transition zone, Prostate-specific antigen density, Clinically significant prostate cancer, Artificial intelligence

## Abstract

**Objectives:**

The study aimed to assess the predictive performance of transition zone PSA density (TZ-PSAD) compared to conventional PSA density (PSAD) in detecting clinically significant prostate cancer (csPCa) among patients with negative pre-biopsy MRI findings.

**Materials and methods:**

The study included 606 patients with negative MRI findings who subsequently underwent transrectal ultrasound-guided systematic biopsy. AI software automatically measured prostate and zonal volumes, from which PSAD and TZ-PSAD (total PSA/transition zone volume) were calculated. Diagnostic performances were evaluated using ROC curve analysis, risk stratification was applied to select patients needing biopsy, and independent predictors of imaging-invisible csPCa were determined through univariate and multivariate analyses.

**Results:**

51 patients (8.4%) were diagnosed with csPCa. TZ-PSAD demonstrated significant superior discriminative ability (AUC = 0.718) compared to PSAD (AUC = 0.686; *p* = 0.019). Patients with TZ-PSAD ≥ 0.35 ng/mL/cc had a csPCa detection rate of 20.1%, while those below this threshold had a rate of 4.1%. The optimal TZ-PSAD threshold of 0.35 ng/mL/cc showed superior performance than the guideline-recommended PSAD threshold of 0.2 ng/mL/cc. Multivariate analysis identified TZ-PSAD as a strong independent predictor of imaging-invisible csPCa.

**Conclusions:**

TZ-PSAD outperforms conventional PSAD in predicting csPCa among men with negative MRI, offering a valuable tool for risk stratification. This facilitates individualized risk assessment, potentially reducing unnecessary biopsies and optimizing patient management.

**Critical relevance statement:**

Our AI system delivers accurate and reproducible prostate zone segmentation, while TZ-PSAD derived from AI outperforms conventional PSAD in detecting csPCa in MRI-negative patients and serves as an effective triage tool to optimize biopsy decision-making and reduce unnecessary systematic biopsies.

**Key Points:**

- Our AI system enables accurate and reproducible segmentation and measurement of prostate zones.
- TZ-PSAD demonstrates significantly superior diagnostic performance over conventional PSAD for identifying men with a negative MRI who will have csPCa on a systematic biopsy.
- TZ-PSAD represents an effective triage metric to reduce unwarranted systematic biopsies in MRI-negative patients.

**Graphical Abstract:**

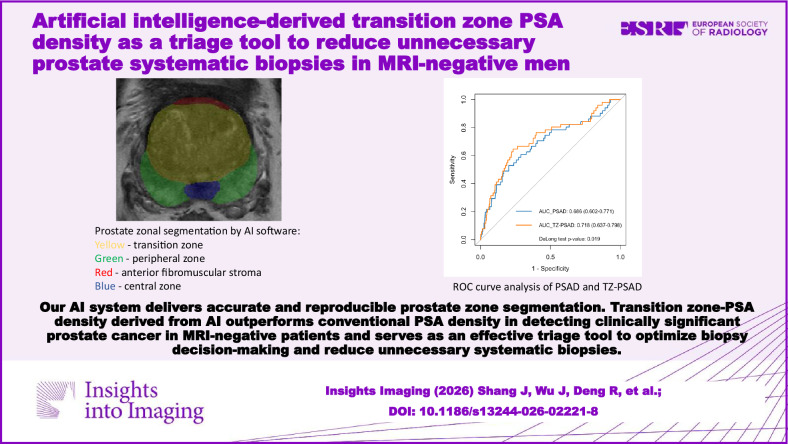

## Introduction

MRI plays a crucial role in prostate cancer screening, with guidelines recommending targeted combined with systematic biopsy for patients presenting with suspicious lesions identified on MRI [1]. However, for individuals with elevated prostate-specific antigen (PSA) levels or positive digital rectal examination (DRE) findings but negative MRI results (Prostate Imaging Reporting and Data System (PI-RADS) v2.1 scores of 1–2), systematic biopsy should still be considered [2]. This strategy, however, lacks precision and may lead to unnecessary biopsies, as only 6% to 13% of patients with negative MRI are ultimately diagnosed with clinically significant prostate cancer (csPCa), indicating that the majority could potentially avoid unnecessary invasive procedures [3–7]. Therefore, accurately distinguishing csPCa from non-csPCa in patients with negative MRI remains a critical but unresolved clinical challenge, with important implications for patient management and healthcare resource optimization.

Benign prostatic hyperplasia (BPH) in the transition zone is a key contributor to elevated PSA levels [8, 9]. For patients with BPH, who often present with enlarged prostate volumes, the risk of unnecessary biopsies due to false-positive PSA findings becomes especially prominent. Previous studies have shown that transition zone PSA density (TZ-PSAD), defined as the ratio of total PSA (tPSA) level to transition zone volume (TZV), outperforms conventional PSA density (PSAD, calculated as tPSA divided by the whole prostate volume) in predicting csPCa [10–13]. However, the diagnostic value of TZ-PSAD specifically within populations with negative MRI remains unclear and requires further investigation. Additionally, BPH leads to an irregular and asymmetrical shape of the transition zone, which poses challenges for accurate volume measurement.

We have developed deep learning-based artificial intelligence (AI) software and integrated it into the structured reporting software to assist radiologists in diagnosing prostate MRI [14]. The AI software enables precise and efficient execution of the following functionalities: prostate segmentation and measurement of prostate diameter and volume; differentiation and measurement of prostate zones; and detection and measurement of csPCa lesions [15–17]. The volume measurement accuracy of the AI software was confirmed to be high based on Bland–Altman analysis in an external multicenter study [18]. Another external multicenter validation study demonstrated that the diagnostic performance of radiologists for csPCa was significantly improved with AI assistance, with negative predictive value (0.947 vs. 0.892, *p* = 0.037) and positive predictive value (0.661 vs. 0.558, *p* = 0.014) compared to radiologists diagnosing independently [19].

In this study, we evaluated the predictive performance of conventional PSAD and TZ-PSAD for csPCa in patients diagnosed as negative MRI by radiologists with AI assistance, aiming to stratify patients by risk and potentially spare low-risk individuals from unnecessary systematic biopsies.

## Materials and methods

### Study design and population

The retrospective study received approval from the institutional review board of Peking University First Hospital (2023-272) and was registered on ClinicalTrials.gov (NCT06842264). The requirement for informed consent was waived due to its design.

We reviewed the clinical data of 2725 patients who underwent prostate MRI due to elevated PSA (≥ 3 ng/mL)^2^ followed by transrectal ultrasound (TRUS)-guided prostate biopsy at our institution from November 2022 to February 2025. The exclusion criteria were as follows: (1) diagnosis of positive MRI by radiologists with AI assistance; (2) biopsy performed more than 3 months after MRI [20, 21]; (3) history of previous prostate biopsy; (4) prior surgical treatment or 5-alpha-reductase inhibitor therapy [22–24] for BPH; (5) history of urinary tract infection or acute urinary retention; (6) presence of urinary or suprapubic catheters at the time of MRI; (7) deficient quality of MRI images; (8) data loss. A total of 606 eligible patients were ultimately enrolled in the study.

### AI software workflow

**Fig. 1:**
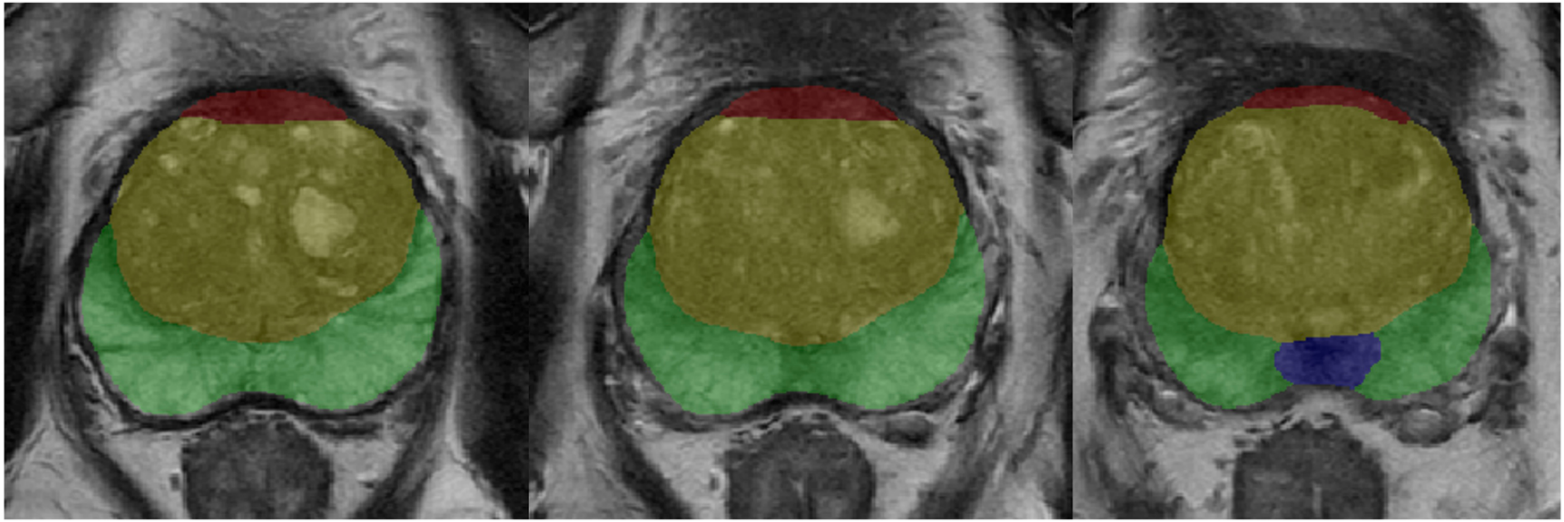
Example of prostate zonal segmentation by the artificial intelligence software in a patient. Yellow: transition zone; green: peripheral zone; red, anterior fibromuscular stroma; blue, central zone

A deep learning-based AI software was developed to assist in the diagnosis of prostate MRI [19, 25]. The software integrates four distinct and sequential models: (1) classification of MRI sequences; (2) prostate gland segmentation and measurement of the prostate volume; (3) segmentation of prostate zones (Fig. 1); (4) identification and measurement of csPCa lesions [19]. These models were implemented in the predefined sequence, and the output results were automatically incorporated into the structured reporting system and presented to radiologists for review [14].

### MRI scanning protocols

Prostate MRI examinations were conducted using 3.0-Tesla scanners (Discovery MR750, GE Healthcare; Ingenia, Philips Healthcare; uMR 790, United Imaging Healthcare) equipped with a pelvic phased-array coil. The imaging protocol included axial T1-weighted imaging (T1WI), axial T2-weighted imaging (T2WI) and axial diffusion-weighted imaging (DWI) with corresponding apparent diffusion coefficient (ADC) maps, as well as sagittal T1WI and coronal T2WI. Axial T2WI and DWI/ADC sequences were utilized for AI analysis, and the corresponding scanning parameters are shown in Table S1.

### AI-assisted MRI interpretation by radiologists [19]

When radiologists opened a case in the reporting system, the AI software had already segmented the prostate and annotated suspicious lesions, providing pre-measured prostate diameters and volume, as well as the location and maximum diameter of each lesion. Radiologists comprehensively reviewed the images and could accept, modify, or reject the AI-generated findings at their discretion. They then evaluated additional findings, such as extra-prostatic extension, invasion of surrounding structures, and other benign abnormalities. Afterward, radiologists summarized the image findings and provided a diagnostic impression. All initial interpretations were subject to secondary review and confirmation by senior-level radiologists.

### Biopsy and pathology procedures

As no suspicious lesions were indicated by the MRI, all patients subsequently underwent a TRUS-guided 12-core systematic prostate biopsy. The TRUS was carried out using color Doppler ultrasound systems (Hi Vision, Hitachi; EPIQ 7, Philips Healthcare).

Biopsy specimens were evaluated by two experienced uropathologists, both with over 15 years of diagnostic experience, in accordance with the 2014 grade system of the International Society of Urological Pathology (ISUP) [26]. csPCa was defined as a Gleason score ≥ 7 [27].

### Data collection

We collected the demographic and clinical data of patients with negative MRI, including age, DRE findings, tPSA level, prostate volume (PV), volumes of the four prostate zones (TZV; peripheral zone volume, PZV; central zone volume, CZV; anterior fibromuscular stroma volume, AFSV), Gleason score, and ISUP grade. PV, TZV, PZV, CZV, and AFSV were automatically measured by the AI software. PSAD was calculated by dividing tPSA by PV, and zone-specific PSADs were calculated by dividing tPSA by the corresponding zonal volumes, including TZ-PSAD (tPSA/TZV), PZ-PSAD (tPSA/PZV), CZ-PSAD (tPSA/CZV), and AFS-PSAD (tPSA/AFSV).

### Statistical analysis

Descriptive analysis was performed for demographic and clinical data. Continuous variables were tested for normality and were presented as mean (standard deviation) for normally distributed data or median (interquartile range) for non-normally distributed data, and categorical variables were summarized as counts and percentages. The chi-square test or Fisher’s exact test was used for analysis of categorical variables, while Student’s *t*-test or Mann–Whitney U test was employed for comparison of continuous variables.

The diagnostic performances of PV, the four zonal volumes, PSAD, and the four zone-specific PSADs for predicting csPCa were evaluated using receiver operating characteristic (ROC) curve analysis. The area under the curve (AUC) was calculated for each predictor. DeLong test was applied to compare the diagnostic performances of the four zonal volumes and zone-specific PSADs with that of PV and PSAD, respectively.

The optimal cut-off values for PV, PSAD, TZV, and TZ-PSAD were determined by Youden’s index. According to these thresholds, stratified csPCa detection rates were calculated and presented for clinical interpretability. Further stratification was also conducted by grouping patients into multiple risk categories based on these predictors.

True Positive (TP), False Positive (FP), True Negative (TN), and False Negative (FN) for the patient population were calculated based on the optimal threshold of TZ-PSAD and the recommended threshold of PSAD (0.2 ng/mL/cc) from the EAU guidelines, as well as the corresponding sensitivity, specificity, positive predictive value, and negative predictive value.

Variables with potential associations (*p* < 0.1) in the univariate analysis were included in the multivariate model for further evaluation. Considering that PSAD and TZ-PSAD represent the ratios of tPSA to PV and TZV, respectively, PV and TZV were excluded from the multivariate model to avoid potential multicollinearity. In addition, moderate multicollinearity was observed (variance inflation factor (VIF) > 5) when both PSAD and TZ-PSAD were included in the model. Therefore, TZ-PSAD was retained in the model instead of PSAD.

All statistical analyses were conducted using R software (version 4.3.3). A *p*-value < 0.05 was considered statistically significant.

## Results

### Patient characteristics

**Fig. 2:**
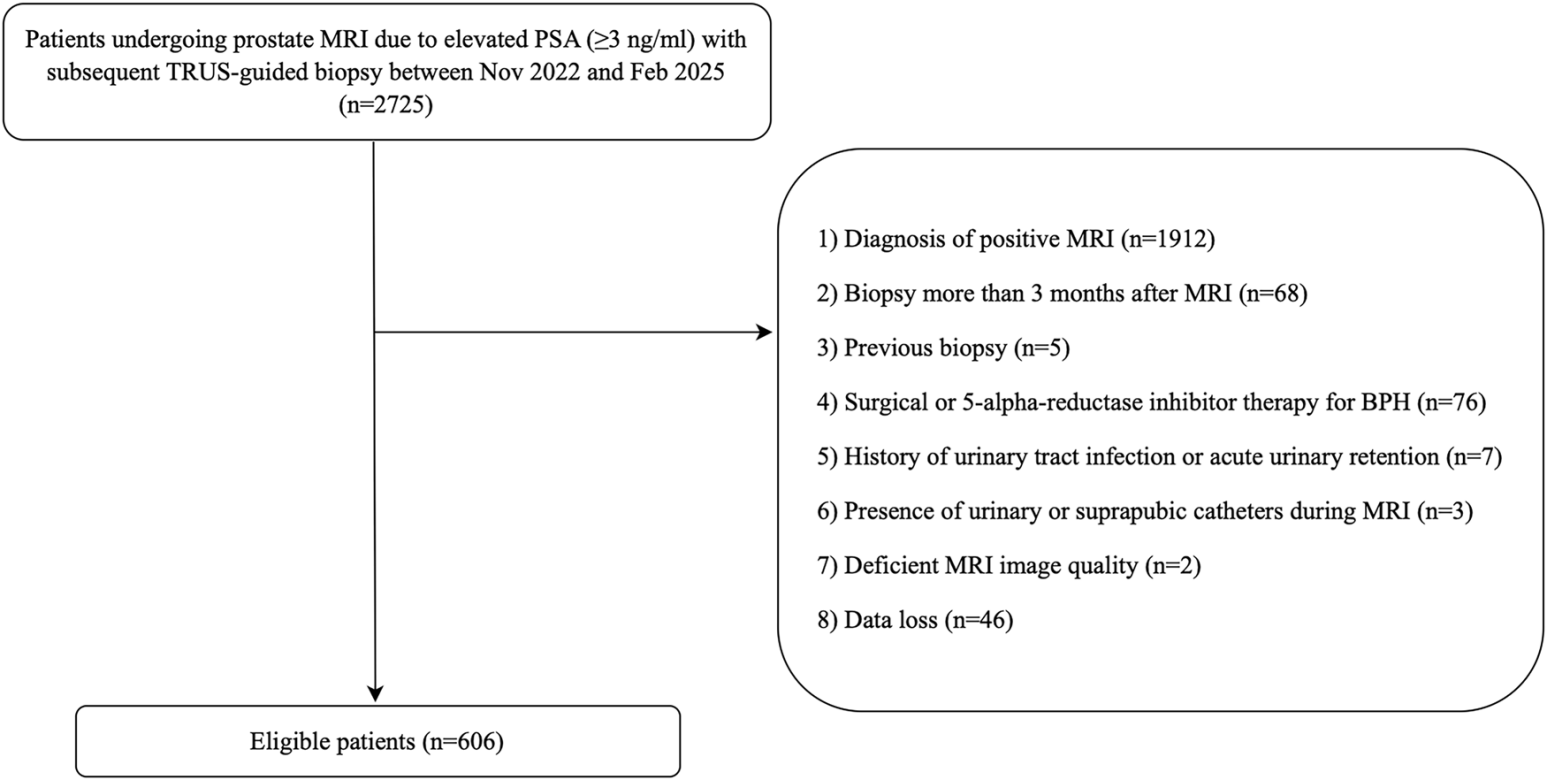
Inclusion and exclusion flowchart of the study population. PSA, prostate-specific antigen; MRI, magnetic resonance imaging; TRUS, transrectal ultrasound; AI, artificial intelligence; BPH, benign prostatic hyperplasia

A total of 606 patients with negative pre-biopsy MRI were included in this study, among whom 51 (8.4%) were diagnosed with csPCa (Fig. 2). The comparison of baseline characteristics of patients with and without csPCa is shown in Table 1.

**Table 1:** Comparison of characteristics between non-csPCa and csPCa patients

Variable [median (IQR)]	All (*n* = 606)	Non-csPCa (*n* = 555)	csPCa (*n* = 51)	*p*-value
Age, years	65 (60−70)	65 (60−69)	69 (63−72)	< 0.001
tPSA, ng/mL	9.65 (7.00−13.62)	9.70 (7.03−13.83)	8.79 (6.09−11.04)	0.093
PV, mL	64.6 (45.7−88.1)	66.5 (48.2−90.6)	40.6 (30.6−55.1)	< 0.001
TZV, mL	43.3 (26.3−68.4)	45.1 (28.3−70.2)	20.8 (13.0−35.0)	< 0.001
PZV, mL	13.1 (10.5–16.4)	13.1 (10.6−16.7)	11.9 (10.0–14.3)	0.041
CZV, mL	3.1 (2.6–3.7)	3.1 (2.6–3.7)	2.9 (2.3–3.3)	0.008
AFSV, mL	1.8 (1.5–2.2)	1.8 (1.5–2.2)	1.6 (1.4–2.1)	0.028
PSAD, ng/mL/cc	0.15 (0.11−0.21)	0.15 (0.11−0.20)	0.22 (0.15−0.31)	< 0.001
TZ-PSAD, ng/mL/cc	0.24 (0.15−0.37)	0.23 (0.14−0.34)	0.42 (0.27−0.66)	< 0.001
PZ-PSAD, ng/mL/cc	0.70 (0.50−1.12)	0.71 (0.50−1.14)	0.66 (0.48−1.03)	0.547
CZ-PSAD, ng/mL/cc	3.09 (2.16–4.47)	3.08 (2.17−4.49)	3.14 (2.14−4.33)	0.864
AFS-PSAD, ng/mL/cc	5.40 (3.75−7.72)	5.45 (3.78−7.70)	4.76 (3.61−8.71)	0.610
Positive DRE*	52 (8.6)	45 (8.1)	7 (13.7)	0.187

*IQR* interquartile range, *csPCa* clinically significant prostate cancer, *tPSA* total prostate-specific antigen, *PV* prostate volume, *TZV* transition zone volume, *PZV* peripheral zone volume, *CZV* central zone volume, *AFSV* anterior fibromuscular stroma volume, *PSAD* prostate-specific antigen density, *DRE* digital rectal examination

* Frequency (percentage)

Patients with csPCa were older, with smaller PV and TZV, as well as higher PSAD and TZ-PSAD. PZV, CZV, and AFSV were also significantly different between the two groups, whereas the corresponding zone-specific PSADs (PZ-PSAD, CZ-PSAD, and AFS-PSAD) did not show statistical significance.

### Diagnostic performance of volumes and PSADs

Table 2 demonstrates the comparison of AUCs across the relevant variables. Among the five volumes, although PV yielded the highest AUC (0.767) for predicting csPCa, the AUC of TZV (0.756) was not significantly different (*p* = 0.190). In contrast, the AUCs of the remaining three zonal volumes were significantly lower than that of PV (all *p* < 0.001).

**Table 2:** AUC with 95% CI of different predictors for diagnosing csPCa and *p*-value of DeLong test compared to PV or PSAD

Predictor	AUC	95% CI	*p*-value
PV	0.767	0.698−0.837	-
TZV	0.756	0.688−0.823	0.190
PZV	0.586	0.514−0.659	< 0.001
CZV	0.612	0.530−0.694	< 0.001
AFSV	0.593	0.505−0.680	< 0.001
PSAD	0.686	0.602−0.771	-
TZ-PSAD	0.718	0.637−0.798	0.019
PZ-PSAD	0.526	0.445−0.606	< 0.001
CZ-PSAD	0.507	0.424−0.590	< 0.001
AFS-PSAD	0.522	0.432−0.612	< 0.001

*AUC* area under the curve, *CI* confidence interval, *PV* prostate volume, *TZV* transition zone volume, *PZV* peripheral zone volume, *CZV* central zone volume, *AFSV* anterior fibromuscular stroma volume, *PSAD* prostate-specific antigen density

When comparing conventional PSAD with the four zone-specific PSADs, TZ-PSAD achieved the highest discriminative ability for csPCa (AUC = 0.718), which was significantly greater than that of conventional PSAD (AUC = 0.686; *p* = 0.019). Conversely, the other three zone-specific PSADs had significantly lower AUCs compared to PSAD (all *p* < 0.001), and their 95% confidence intervals all crossed 0.5, indicating minimal predictive value for csPCa.

### Risk stratification of csPCa

Given that TZV exhibited a comparable predictive performance to PV for csPCa, and TZ-PSAD outperformed conventional PSAD, patients were stratified into two risk levels for csPCa based on the optimal diagnostic thresholds of these four predictors based on the optimal diagnostic thresholds of these four predictors (Table 3).

**Table 3:** Diagnostic performance of csPCa predictors and stratified detection rates

Predictor	Threshold	Stratification	csPCa detection rate
PV, mL	45.9	> 45.9	3.8% (17/451)
		≤ 45.9	21.9% (34/155)
TZV, mL	34.5	> 34.5	3.4% (13/379)
		≤ 34.5	16.7% (38/227)
PSAD, ng/mL/cc	0.22	< 0.22	5.5% (26/471)
		≥ 0.22	18.5% (25/135)
TZ-PSAD, ng/mL/cc	0.35	< 0.35	4.1% (18/442)
		≥ 0.35	20.1% (33/164)

*csPCa* clinically significant prostate cancer, *PV* prostate volume, *TZV* transition zone volume, *PSAD* prostate-specific antigen density

Among patients with PV > 45.9 mL, the csPCa detection rate was 3.8%. In contrast, patients with PV ≤ 45.9 mL showed a csPCa detection rate of 21.9%. Similarly, patients with TZV > 34.5 mL, PSAD < 0.22 ng/mL/cc, or TZ-PSAD < 0.35 ng/mL/cc had csPCa detection rates of 3.4%, 5.5%, and 4.1%, respectively, whereas those below or above these cutoffs had detection rates exceeding 10%.

To further explore the stratified predictive value of the four key indicators, we performed a multi-level risk stratification. As shown in Table 4, the detection rate of csPCa increased notably as PV and TZV decreased, or as PSAD and TZ-PSAD increased. For all four predictors, in the highest-risk groups, defined by PV ≤ 46 mL, TZV ≤ 35 mL, PSAD ≥ 0.20 ng/mL/cc, and TZ-PSAD ≥ 0.35 ng/mL/cc, detection rates of csPCa reached up to 21.8%, 16.4%, 16.5%, and 20.1%, respectively.

**Table 4:** csPCa detection rates stratified by PV, TZV, PSAD, and TZ-PSAD risk groups

Predictor	Risk groups	csPCa detection rate
PV, mL	PV > 86	1.9% (3/158)
	66 < PV ≤ 86	3.8% (5/131)
	46 < PV ≤ 66	5.6% (9/161)
	PV ≤ 46	21.8% (34/156)
TZV, mL	TZV > 65	1.8% (3/170)
	50 < TZV ≤ 65	4.3% (4/92)
	35 < TZV ≤ 50	5.4% (6/112)
	TZV ≤ 35	16.4% (38/232)
PSAD, ng/mL/cc	PSAD < 0.10	5.5% (7/127)
	0.10 ≤ PSAD < 0.15	3.1% (5/162)
	0.15 ≤ PSAD < 0.20	7.5% (11/147)
	PSAD ≥ 0.20	16.5% (28/170)
TZ-PSAD, ng/mL/cc	TZ-PSAD < 0.15	5.2% (8/155)
	0.15 ≤ TZ-PSAD < 0.25	2.3% (4/173)
	0.25 ≤ TZ-PSAD < 0.35	5.3% (6/114)
	TZ-PSAD ≥ 0.35	20.1% (33/164)

*csPCa* clinically significant prostate cancer, *PV* prostate volume, *TZV* transition zone volume, *PSAD* prostate-specific antigen density

### Diagnostic performance comparison for TZ-PSAD and PSAD thresholds

Compared to the PSAD threshold of 0.2 ng/mL/cc recommended by EAU guidelines, the optimal TZ-PSAD threshold of 0.35 ng/mL/cc resulted in a higher number of true positive cases (TP) and true negative cases (TN), as well as fewer false-positive cases (FP) and false-negative cases (FN) (Table S2). The sensitivity, specificity, positive predictive value (PPV), and negative predictive value (NPV) of TZ-PSAD were all superior to those of PSAD.

### Univariate and multivariate analyses

Univariate analysis revealed that age, PV, TZV, PSAD, and TZ-PSAD were all significantly associated with csPCa (all *p* < 0.001) (Table 5). Among these, smaller PV and TZV, as well as higher PSAD and TZ-PSAD values, were correlated with an increased likelihood of csPCa. After variable selection, age and TZ-PSAD were ultimately included in the multivariate analysis. Multivariate logistic regression demonstrated that both age (OR (95% CI): 1.116 (1.069–1.168), *p* < 0.001) and TZ-PSAD (OR (95% CI): 8.780 (4.025–21.768), *p* < 0.001) were strong independent predictors of csPCa.

**Table 5:** Univariate and multivariate logistic regression for csPCa independent predictor selection

Variable	Univariate		Multivariate	
	OR (95% CI)	*p*-value	OR (95% CI)	*p*-value
Age, years	1.082 (1.038−1.128)	< 0.001	1.116 (1.069–1.168)	< 0.001
Positive DRE	1.803 (0.709−4.010)	0.176		
tPSA, ng/mL	0.968 (0.914−1.017)	0.238		
PV, mL	0.957 (0.941−0.972)	< 0.001		
TZV, mL	0.959 (0.943−0.974)	< 0.001		
PSAD, ng/mL/cc	51.520 (8.019−397.199)	< 0.001		
TZ-PSAD, ng/mL/cc	4.713 (2.418−10.017)	< 0.001	8.780 (4.025–21.768)	< 0.001

*csPCa* clinically significant prostate cancer, *OR* odds ratio, *tPSA* total prostate-specific antigen, *PV* prostate volume, *TZV* transition zone volume, *PSAD* prostate-specific antigen density, *DRE* digital rectal examination

## Discussion

Given that MRI may miss a portion of imaging-invisible but clinically significant lesions [28], risk prediction and stratification in this population remain a major clinical challenge. In this study, we revealed that TZ-PSAD significantly outperforms conventional PSAD and other zone-specific PSADs in predicting csPCa among men with negative pre-biopsy MRI, providing a practical tool to guide biopsy decisions and potentially reduce unnecessary procedures.

TZ-PSAD demonstrated significantly better discriminative ability for csPCa than conventional PSAD (AUC: 0.718 vs. 0.686, *p* = 0.019). This superiority suggests that incorporating transition zone–adjusted PSA density may improve risk assessment in men with negative MRI, where conventional PSAD may misestimate disease probability. TZ-PSAD appears to more accurately reflect tumor burden by adjusting PSA levels according to the glandular zone where most benign hyperplasia occurs.

Risk stratification revealed that the csPCa detection rate was only 3.8% among patients with TZ-PSAD < 0.35 ng/mL/cc, which is below the 10% biopsy-free threshold recommended by the EAU guidelines [2], indicating that biopsy may be safely avoided in this subgroup. In contrast, patients with TZ-PSAD ≥ 0.35 ng/mL/cc showed a markedly higher csPCa detection rate of 21.9%, suggesting a greater likelihood of clinically significant disease and warranting consideration of biopsy. The multi-level risk stratification analysis demonstrated consistent results, with csPCa detection rates of 5.2%, 2.3%, and 5.3% across the first three risk groups, all remaining below the commonly accepted biopsy threshold. These findings underscore the ability of TZ-PSAD to provide clinically meaningful risk stratification, facilitating the identification of patients who may benefit from biopsy and those who can be safely monitored.

The EAU guidelines recommend that in patients without a family history of prostate cancer, with PSAD < 0.2 ng/mL/cc, negative DRE, and negative MRI findings, PSA monitoring may be considered as an alternative to systematic biopsy [2]. Consistent with this recommendation, our study identified an optimal PSAD threshold of 0.22 ng/mL/cc for detecting csPCa in MRI-negative men. Among those with PSAD < 0.2 ng/mL/cc, the csPCa detection rate was 5.3%, below the 10% biopsy-free threshold, whereas it increased to 16.5% in patients with PSAD ≥ 0.2 ng/mL/cc, supporting biopsy in this subgroup.

Our study confirms that TZ-PSAD significantly outperforms conventional PSAD in predicting csPCa among men with negative pre-biopsy MRI results. The finding is consistent with recent studies that have investigated the diagnostic role of TZ-PSAD in prostate cancer detection and reported superior predictive value compared to PSAD [10–13, 29]. Specifically, both Kuanar et al [12] and Esengur et al [10] confirmed that TZ-PSAD achieved higher diagnostic accuracy than PSAD for predicting csPCa, which is in agreement with our results. Furthermore, Studies by Jin et al [11] and Castro et al [13] supported the advantage of TZ-PSAD in distinct clinical contexts, including patients with PI-RADS 3 lesions and with PSA levels between 2.6 and 10 ng/mL, further reinforcing the robustness of our findings across different populations.

However, our study differs from previous research in two key aspects. First, most existing studies estimated prostate and transition zone volumes using the ellipsoid formula, which assumed a regular gland shape. Because the prostate and its subzones can be anatomically irregular [30], this geometric approximation may introduce a degree of measurement variability. In our study, the AI-based system developed and externally validated with multicenter datasets was applied to obtain volumetric measurements with higher precision than the ellipsoid formula. This methodological refinement provides more consistent volume measurement and helps reduce potential errors in density calculation. Second, previous studies either focused on unselected populations undergoing MRI and biopsy or, when limited to MRI-negative cohorts, have not investigated the role of TZ-PSAD in this specific clinical context [4–7, 31–33]. Considering that transition zone hyperplasia is a major contributor to PSA elevation in MRI-negative men, our study introduced the assessment of TZ-PSAD for predicting imaging-invisible csPCa. This approach offers a more physiologically relevant biomarker that better reflects PSA elevation secondary to transition zone enlargement.

The clinical value of TZ-PSAD becomes particularly apparent in patients with negative pre-biopsy MRI findings, a group in which the possibility of missing imaging-invisible but clinically significant lesions remains a persistent challenge. Reliance on conventional PSAD in this context may lead to overtreatment of benign conditions. In our study, TZ-PSAD demonstrated strong diagnostic performance, with an optimal threshold of 0.35 ng/mL/cc yielding notably better discrimination than PSAD. By enabling more accurate risk stratification that does not depend solely on MRI visibility, TZ-PSAD provides a valuable adjunct to clinical decision-making. It helps identify patients with truly low risk who may safely avoid unnecessary intervention, while also flagging those who warrant further diagnostic evaluation despite negative imaging. Ultimately, integrating TZ-PSAD into routine assessment could reduce overdiagnosis and overtreatment, minimize patient anxiety, and support more efficient use of healthcare resources in prostate cancer screening and management.

This study has several limitations. First, it was a retrospective single-center study with a relatively homogeneous patient population, which may restrict the generalizability of the findings to more diverse clinical settings. Second, while the diagnostic performance of TZ-PSAD was systematically evaluated, its clinical utility has yet to be externally validated in independent cohorts, which underscores important directions for future research. To address this, we plan to collaborate with other centers for external validation and conduct multicenter prospective studies. In addition, integrating radiomic features or molecular biomarkers with TZ-PSAD may further improve the accuracy and robustness of csPCa risk stratification in patients with negative MRI.

## Conclusions

TZ-PSAD demonstrated superior diagnostic performance over conventional PSAD in men with negative pre-biopsy MRI, offering an effective triage tool for identifying imaging-invisible csPCa. These findings highlight its clinical value in optimizing biopsy decision-making and reducing unnecessary procedures.

## ELECTRONIC SUPPLEMENTARY MATERIAL

Supplementary information

## Data Availability

The data analyzed during the current study are not publicly available due to institutional regulations, but are available from the corresponding author on reasonable request.

## Abbreviations

ADC: Apparent diffusion coefficient
AFSV: Anterior fibromuscular stroma volume
AI: Artificial intelligence
AUC: Area under the curve
BPH: Benign prostatic hyperplasia
csPCa: Clinically significant prostate cancer
CZV: Central zone volume
DRE: Digital rectal examination
DWI: Diffusion-weighted imaging
FN: False negative
FP: False positive
ISUP: International Society of Urological Pathology
MRI: Magnetic resonance imaging
NPV: Negative predictive value
PI-RADS: Prostate Imaging Reporting and Data System
PPV: Positive predictive value
PSA: Prostate-specific antigen
PSAD: PSA density
PV: Prostate volume
PZV: Peripheral zone volume
ROC: Receiver operating characteristic
T1WI: T1-weighted imaging
T2WI: T2-weighted imaging
TN: True negative
TP: True positive
tPSA: Total PSA
TRUS: Transrectal ultrasound
TZ-PSAD: Transition zone PSA density
TZV: Transition zone volume
VIF: Variance inflation factor
