# Supplementary material for: Artificial intelligence-derived transition zone PSA density as a triage tool to reduce unnecessary prostate systematic biopsies in MRI-negative men
Source: Insights Imaging. 2026 Feb 10;17:40. doi: 10.1186/s13244-026-02221-8 (PMC12891307; doi:10.1186/s13244-026-02221-8)
Supplement: Supplementary file 1 — Supplementary information [file 13244_2026_2221_MOESM1_ESM.pdf]

Artificial intelligence-derived transition zone PSA density as a triage tool to reduce unnecessary prostate systematic biopsies in MRI-negative men
ELECTRONIC SUPPLEMENTARY MATERIAL

Table S1. The scanning parameters of MRI sequences used for AI analysis

	Sequences	
	**T2WI**	**DWI**
B value, s/mm^2	-	0, 1400
Repetition time, ms	3130 [3000, 3680]	2950 [2630, 4260]
Echo time, ms	92 [88, 115]	62 [60, 64]
Pixel bandwidth, MHz	163 [163, 218]	1950 [1560, 1950]
Slice thickness, mm	4.0 [4.0, 4.0]	4.0[4.0, 4.0]
Slice spacing, mm	4.0 [4.0, 4.0]	4.0[4.0, 4.5]
Pixel spacing, mm	0.469 [0.391, 0.469]	0.938 [0.938, 1.30]
Reconstruction diameter, mm	240 [220, 240]	240 [200, 240]

Values are presented as median [interquartile range], with the B value represented by the minimum and maximum values.

Table S2 Comparison of diagnostic performances for TZ-PSAD and guideline-recommended PSAD thresholds

	**TZ-PSAD (0.35 ng/ml/cc)**	**PSAD (0.2 ng/ml/cc)**
TP	33	28
FP	131	142
TN	424	413
FN	18	23
sensitivity	0.647	0.549
specificity	0.764	0.744
PPV	0.201	0.165
NPV	0.959	0.947

Abbreviations: TZ, transition zone; PSAD, prostate specific antigen density; TP, true positive; FP, false positive; TN, true negative; FN, false negative; PPV, positive predictive value; NPV, negative predictive value.
